# Sequential algorithm to stratify liver fibrosis risk in overweight/obese metabolic dysfunction-associated fatty liver disease

**DOI:** 10.3389/fendo.2022.1056562

**Published:** 2023-01-06

**Authors:** Chi-Ho Lee, David Tak-Wai Lui, Raymond Hang-Wun Li, Michele Mae-Ann Yuen, Carol Ho-Yi Fong, Ambrose Pak-Wah Leung, Justin Chiu-Man Chu, Loey Lung-Yi Mak, Tai-Hing Lam, Jean Woo, Yu-Cho Woo, Aimin Xu, Hung-Fat Tse, Kathryn Choon-Beng Tan, Bernard Man-Yung Cheung, Man-Fung Yuen, Karen Siu-Ling Lam

**Affiliations:** ^1^ Department of Medicine, School of Clinical Medicine, University of Hong Kong, Queen Mary Hospital, Hong Kong, Hong Kong SAR, China; ^2^ State Key Laboratory of Pharmaceutical Biotechnology, University of Hong Kong, Hong Kong, Hong Kong SAR, China; ^3^ Department of Obstetrics and Gynaecology, University of Hong Kong, Queen Mary Hospital, Hong Kong, Hong Kong SAR, China; ^4^ The School of Public Health, University of Hong Kong, Queen Mary Hospital, Hong Kong, Hong Kong SAR, China; ^5^ Department of Medicine and Therapeutics, The Chinese University of Hong Kong, Hong Kong, Hong Kong SAR, China; ^6^ State Key Laboratory of Liver Research, University of Hong Kong, Hong Kong, Hong Kong SAR, China

**Keywords:** obesity, MAFLD (metabolic associated fatty liver disease), overweight, fatty liver disease, population based study

## Abstract

**Background:**

Non-diabetic overweight/obese metabolic dysfunction-associated fatty liver disease (MAFLD) represents the largest subgroup with heterogeneous liver fibrosis risk. Metabolic dysfunction promotes liver fibrosis. Here, we investigated whether incorporating additional metabolic risk factors into clinical evaluation improved liver fibrosis risk stratification among individuals with non-diabetic overweight/obese MAFLD.

**Materials and methods:**

Comprehensive metabolic evaluation including 75-gram oral glucose tolerance test was performed in over 1000 participants from the New Hong Kong Cardiovascular Risk Factor Prevalence Study (HK-NCRISPS), a contemporary population-based study of HK Chinese. Hepatic steatosis and fibrosis were evaluated based on controlled attenuation parameter and liver stiffness (LS) measured using vibration-controlled transient elastography, respectively. Clinically significant liver fibrosis was defined as LS ≥8.0 kPa. Our findings were validated in an independent pooled cohort comprising individuals with obesity and/or polycystic ovarian syndrome.

**Results:**

Of the 1020 recruited community-dwelling individuals, 312 (30.6%) had non-diabetic overweight/obese MAFLD. Among them, 6.4% had LS ≥8.0 kPa. In multivariable stepwise logistic regression analysis, abnormal serum aspartate aminotransferase (AST) (OR 7.95, p<0.001) and homeostasis model assessment of insulin resistance (HOMA-IR) ≥2.5 (OR 5.01, p=0.008) were independently associated with LS ≥8.0 kPa, in a model also consisting of other metabolic risk factors including central adiposity, hypertension, dyslipidaemia and prediabetes. A sequential screening algorithm using abnormal AST, followed by elevated HOMA-IR, was developed to identify individuals with LS ≥8.0 kPa, and externally validated with satisfactory sensitivity (>80%) and negative predictive value (>90%).

**Conclusion:**

A sequential algorithm incorporating AST and HOMA-IR levels improves fibrosis risk stratification among non-diabetic overweight/obese MAFLD individuals.

## Introduction

Metabolic dysfunction-associated fatty liver disease (MAFLD) is a new nomenclature defining fatty liver disease with metabolic dysfunction proposed by an international expert panel in 2020, and affects one-third of the global adult population (1–3). MAFLD can be classified into three subtypes, based on the presence of hepatic steatosis co-existing with any one of the following including (1) type 2 diabetes (T2D-MAFLD); (2) overweight or obesity (overweight/obese MAFLD), defined as body mass index (BMI) ≥23kg/m^2^ in Asians and 25kg/m^2^ in Caucasians; and (3) in lean/normal weight individuals, the presence of any two of the other evidence of metabolic dysfunction (Lean MAFLD), including central obesity, elevated blood pressure (BP), dyslipidaemia, prediabetes, increased homeostasis model assessment of insulin resistance (HOMA-IR), and elevated circulating high-sensitivity C-reactive protein (hsCRP) levels (1).

In fatty liver disease, liver fibrosis is the most important determinant of adverse long-term outcomes. Higher stage of liver fibrosis is associated with all-cause, as well as liver-related, mortality and morbidity (4, 5). Early identification of individuals with clinically significant liver fibrosis, who are at risk of compensated advanced chronic liver disease (cACLD) (6, 7), is important to facilitate more targeted follow-up and surveillance, especially among overweight and obese individuals in whom the prevalence of MAFLD is over 50% (8). Several recent studies have suggested that the prevalence and risk of liver fibrosis differ across the three MAFLD subtypes (9–11). However, while individuals with overweight/obese MAFLD constitute the largest subgroup within the MAFLD population, no report thus far has evaluated the optimal strategy for stratifying liver fibrosis risk in these individuals. Previous studies in non-alcoholic fatty liver disease (NAFLD) have shown that the presence of metabolic dysfunction was closely associated with the development of liver fibrosis (12). Hence, we investigated whether incorporating additional metabolic risk factors into clinical evaluation would improve liver fibrosis risk stratification among individuals with overweight/obese MAFLD, using a contemporary population-based study of Hong Kong (HK) Chinese with comprehensive metabolic assessment.

## Materials and methods

### Study participants

All participants were recruited from the New HK Cardiovascular Risk Factor Prevalence Study (NCRISPS), an ongoing population-based, cross-sectional study established since December 2019, to determine the updated sex and age-stratified prevalence of cardiovascular risk factors, cardiovascular diseases (CVD) and related disorders in HK Chinese. The protocol was similar to the previously published HK Cardiovascular Risk Factor Prevalence Study (CRISPS) (13). Individuals aged 25 – 74 years were recruited from the community through systematic sampling of representative replicates of living quarters in HK, obtained from the Census and Statistics Department of the HK Special Administrative Region. Pregnant women, individuals with physical or mental illness which precluded them from travelling to the study centre for health assessment, or from providing informed consent, were excluded. The protocol of NCRISPS was approved by the Ethics Committee of the University of Hong Kong/Hospital Authority Hong Kong West Cluster (IRB Ref: UW-18-610). Written informed consent was obtained from all participants before any study-related procedures.

### Clinical assessments

All NCRISPS participants attended the study visit after an overnight fast of at least 8 hours. Each participant completed a detailed questionnaire which included demographics (age, sex, occupation, income, education level, smoking and alcohol intake), family history, medical (personal history of diabetes, hypertension, hyperlipidaemia, CVD, cancers, chronic liver diseases in particular viral hepatitis, Wilson’s disease, alpha-1 antitrypsin deficiency, autoimmune hepatitis and primary biliary cholangitis), and drug histories (anti-diabetic, lipid lowering, anti-hypertensive, and steatogenic medications such as amiodarone, tamoxifen, methotrexate etc.). Bloods were drawn for complete blood count and serum creatinine levels. Estimated glomerular filtration rate (eGFR) of the participants was calculated using the Chronic Kidney Disease Epidemiology Collaboration (CKD-EPI) equation as described previously (14).

### Metabolic assessments

Anthropometric parameters including body weight (BW), height (BH), BMI, waist and hip circumferences (WC and HC, respectively) and BP were measured as previously described (13). Fasting bloods were drawn for glycated haemoglobin (HbA1c) and lipid profile. All participants, except those on anti-diabetic medications, underwent a 75-gram oral glucose tolerance test (OGTT). Moreover, except for those receiving insulin therapy, fasting insulin level was measured to determine the HOMA-IR level (15).

### Hepatic assessments

Liver biochemistry including serum levels of alanine aminotransferase (ALT) and aspartate aminotransferase (AST) were measured at the Pathology Department of Queen Mary Hospital, Hong Kong, a laboratory accredited by the College of American Pathologists. The definitions of abnormal ALT and AST levels in the laboratory were based on age- and sex-specific reference ranges established using data collected from a cohort of local healthy Chinese (13). Hepatitis B surface antigen (HBsAg) were measured in all participants, whereas antibody against hepatitis C virus (Anti-HCV) was measured in those with elevated ALT or AST levels above the upper normal range (ALT: 58U/L for men, and 36 U/L for women aged ≤50 years and 45 U/L for women aged >50 years; AST: 38 U/L for men, and 30 U/L for women aged ≤50 years and 37 U/L for women aged >50 years), and/or HBsAg-positivity. Two commonly used non-invasive conventional fibrosis scores including Fibrosis-4 index (FIB-4) and NAFLD fibrosis score (NFS) were determined using published formulae (16).

During the study visit, all participants underwent vibration controlled transient elastography (VCTE) assessments, performed by trained operators using Fibroscan (Echosens, Paris, France) as per protocol described previously (17). In all participants, M probe was used during assessments, unless prompted by the VCTE machine. Controlled attenuation parameter (CAP) and liver stiffness (LS), which assessed the severity of liver steatosis and fibrosis, respectively, were measured with values represented by the median of 10 reliable measurements, defined when the interquartile range was <30% and the success rate was >60%. Only CAP values with an inter-quartile range of 40 dB/m were used to ensure data validity.

### Definition of outcomes and clinical variables

Hepatic steatosis was defined as CAP ≥248 dB/m. Liver fibrosis was graded by LS cut-offs: <8.0 kPa (low risk), 8.0 – 9.5 kPa (intermediate to high risk), ≥9.6 kPa (advanced fibrosis and cirrhosis) (18). In this study, clinically significant liver fibrosis was defined as LS ≥8.0 kPa (7).

In this study consisting of exclusively HK Chinese, overweight and obesity were defined as BMI ≥23kg/m^2^ and ≥27.5kg/m^2^, respectively (1, 19). Central obesity was defined as WC ≥90cm in men and ≥80cm in women (1, 20). Hypertension was defined as BP ≥140/90mmHg or on anti-hypertensive medications. Dyslipidaemia was defined as fasting triglycerides (TG) ≥1.7 mmol/L, high density lipoprotein-cholesterol (HDL-C) <1.3 mmol/L in women and <1.0 mmol/L in men, low density lipoprotein-cholesterol (LDL-C) ≥3.4 mmol/L, or on lipid-lowering medications. Normal glucose tolerance (NGT) was defined as FG <5.6 mmol/L and 2-hour blood glucose (2hG) <7.8 mmol/L. IFG was defined as FG ≥5.6 mmol/L and <7.0 mmol/L, whereas IGT was defined as 2hG ≥7.8 mmol/l and <11.1 mmol/L. Prediabetes included IFG, IGT or elevated HbA1c ≥5.7% and <6.5%. Type 2 diabetes was defined as the presence of any two of the following biochemical abnormalities: FG ≥7.0 mmol/L, or 2hG ≥11.1 mmol/L on OGTT, or HbA1c ≥6.5%, or on anti-diabetic medications (21). CKD was defined as eGFR <60ml/min/1.73m^2^. CVD was defined as any self-reported or medical history of cardiovascular event, including myocardial infarction, stroke, transient ischaemic attack, peripheral vascular disease, heart failure, recorded based on diagnostic codes (402, 404, 410-414,425-447, and 518.4) from the HK Hospital Authority database. Excessive alcohol intake was defined as daily alcohol consumption of >3 drinks in men and >2 drinks in women (1).

### Independent cohorts for external validation

Two independent cohorts consisting of individuals without type 2 diabetes and fulfilled the diagnostic criteria of overweight/obese MAFLD, based on reliable M probe measurements with VCTE, were used to form a pooled external cohort for validating our findings. The first cohort comprised individuals from the Obesity Clinic of Queen Mary Hospital, HK (N=40), whereas the second cohort involved participants from a longitudinal follow-up study of polycystic ovarian syndrome (PCOS) at Queen Mary Hospital, HK (N=31) (22).

### Statistical analysis

All data were analysed with IBM SPSS Statistics 26.0 (http://www.IBM.com/SPSS). Data normality was determined by the Kolmogorov-Smirnov test. Values were reported as mean ± standard deviation (SD), medians with interquartile range (IQR), or percentages, as appropriate. Continuous variables between two groups were compared using independent *t*-test or Mann-Whitney U test, whereas one-way analysis of variance (ANOVA) or Kruskal-Wallis test were used to compare among multiple groups. Categorical variables were compared using Chi-square or Fisher Exact test, as appropriate. Bonferroni correction was applied for multiple comparisons. Cochran-Armitage test was applied for evaluating trend for binary variables, whereas ANOVA linear test or Jonckheere-Terpstra test was used for evaluating continuous variables. Multiple quantile regression analysis was conducted to investigate the associations of clinical variables with LS, accounting for the potential heterogeneity in the association of differently explanatory variables across the different quantiles of LS. Multivariable stepwise logistic regression analysis was performed to evaluate the independent determinants of the presence of LS ≥8.0 kPa and develop a screening algorithm for identifying individuals with overweight/obese MAFLD who had LS ≥8.0 kPa. Sensitivity, specificity, positive and negative predictive values (PPV and NPV) and the area under the receiver operating characteristic curve (AUROC) of the screening algorithm were evaluated to determine its performance. In all statistical tests, a two-sided p-value of <0.05 was considered significant.

## Results

### Overweight/obese MAFLD constituted the largest MAFLD subgroup

Of 1020 NCRISPS participants, 445 (43.6%) of them had fatty liver disease as defined by CAP ≥248 dB/m. All participants had valid CAP and LS measurements on VCTE using M probe. Participants who had fatty liver disease were significantly older (56.1 vs. 54.1 years) (p=0.004), being men (53.7% vs 40.9%) (p<0.001) and ever-smoker (28.1% vs. 20.7%) (p=0.006), with higher BMI (26.4 kg/m^2^ vs. 22.3 kg/m^2^), HOMA-IR (2.31 vs. 1.27) and prevalence of hypertension (43.4% vs. 25.7%), diabetes (19.1% vs 7.0%), dyslipidaemia (74.4% vs. 47.3%) (all p<0.001) and CKD (2.9% vs. 0.9%) (p=0.014) than those who did not. Among these 445 participants, 427 fulfilled the diagnostic criteria of MAFLD, and the majority (73%) had overweight/obese MAFLD, followed by T2D-MAFLD (20%) and lean-MAFLD (7%). Notably, participants with T2D-MAFLD, as compared to the other two subgroups, had significantly higher NFS (p=0.002), CAP (p<0.001) and LS measurements (p<0.001) despite similar serum ALT levels (**Table 1**).

**Table 1:** Clinical characteristics of participants with MAFLD stratified by subtypes (N=427).

	Overweight/obese MAFLD	T2D-MAFLD	Lean-MAFLD	p-value
N	312	85	30	–
Men	175 (56.1%)	42 (49.4%)	15 (50%)	0.49
Age, years	54.5 ± 11.2	61.8 ± 8.4	57.5 ± 8.40	**<0.001**
Ever smoker	94 (30.1%)	22 (25.9%)	6 (20%)	0.42
Current drinker	76 (24.6%)	12 (14.1%)	7 (25.9%)	0.11
Excessive alcohol intake	2	0	0	1.00
BMI, kg/m^2^	26.9 ± 3.0	27.2 ± 4.1	21.6 ± 0.8^***^	**<0.001**
BMI ≥ 27.5 kg/m^2^	111 (35.6%)	34 (40%)	0	**<0.001**
Central obesity	221 (70.8%)	57 (67.1%)	12 (40%)^*^	**0.003**
Prediabetes based on OGTT and/or HbA1c	205 (65.7%)	0	21 (70.0%)	**<0.001**
FG and/or HbA1c	179 (57.4%)	0	17 (56.7%)	**<0.001**
Hypertension	126 (40.4%)^***^	61 (71.8%)	13 (43.3%)^*^	**<0.001**
Dyslipidaemia	228 (73.1%)^*^	75 (88.2%)	24 (80%)	**0.01**
CKD	6 (1.9%)	6 (7.1%)	1 (3.7%)	0.05
CAD	9 (2.9%)^*^	8 (9.4%)	4 (13.3%)	**0.004**
Stroke	6 (60%)	3 (30%)	1 (3.3%)	0.41
Cancer	16 (5.1%)	5 (5.9%)	1 (3.3%)	0.93
Viral hepatitis B or C	17 (5.4%)	6 (7.1%)	2 (6.7%)	0.72
ALT, U/L	26 (19-37)	26 (19-33)	22 (13-33)	0.17
AST, U/L	25 (21-29)^*^	22 (20-27)	24 (20-30)	**0.03**
FIB4	1.04 (0.75-1.37)	1.12 (0.86-1.29)	1.13 (0.92-1.62)	0.23
NFS	-1.86 ± 1.25^**^	-1.37 ± 1.06	-2.00 ± 1.05^*^	**0.002**
CAP, dB/m	296 ± 34^*^	308 ± 37	276 ± 28^c^	**<0.001**
LS, kPa	5.0 (4.3-5.8)^*^	5.4 (4.4-6.7)	4.4 (4.0-4.8)^*^	**<0.001**
LS category				**0.002**
LS ≥ 8.0 kPa	20 (6.4%)^**^	14 (16.5%)	2 (6.7%)	**0.011**
LS ≥ 9.6 kPa	8 (2.6%)	6 (7.1%)	2 (6.7%)	0.33

Values expressed as mean ± standard deviation or median (25^th^ – 75^th^ percentile) or numbers (%). Bonferroni correction was applied for multiple comparisons; *, p<0.05; **, p<0.01; ***, p<0.001 vs. T2D-MAFLD as the reference group.

None of the participants had other chronic liver diseases or use of steatogenic medications.

MAFLD, metabolic dysfunction-associated fatty liver disease; T2D, type 2 diabetes; BMI, body mass index; OGTT, oral glucose tolerance test; HbA1c, glycated haemoglobin; FG, fasting glucose; CKD, chronic kidney disease; CAD, coronary artery disease; ALT, alanine aminotransferase; AST, aspartate aminotransferase; FIB-4, Fibrosis-4 index; NFS, non-alcoholic fatty liver disease fibrosis score; CAP, controlled attenuation parameter; LS, liver stiffness.

Bold values are those with statistical significance.

### Associations of metabolic risk factors with liver fibrosis risk in overweight/obese MAFLD

Among the 312 participants who did not have diabetes and had overweight/obese MAFLD, the majority (93.6%) were at low risk of liver fibrosis with LS <8.0 kPa. (**Table 2**) Higher stages of liver fibrosis were significantly associated with higher serum ALT (p for trend <0.001) and AST levels (p for trend <0.001), CAP (p for trend = 0.002) and NFS (p for trend =0.002). Moreover, with regard to the metabolic risk factors considered in the MAFLD definition (1), participants with higher stages of liver fibrosis had significantly higher prevalence of prediabetes based on OGTT and/or HbA1c (p for trend = 0.01), and were more insulin resistant as indicated by HOMA-IR ≥2.5 (p for trend <0.001). An increasing number of these metabolic risk factors was significantly associated with higher LS values (p for trend <0.001) (**Table 2**).

**Table 2:** Associations of clinical characteristics with the severity of liver fibrosis among participants with overweight/obese MAFLD (N=312).

	LS <8.0 kPa	LS 8.0 – 9.5 kPa	LS ≥9.6 kPa	p for trend
N	292	12	8	–
Men	162 (55.5%)	9 (75.0%)	4 (50.0%)	0.69
Age, years	54.6 ± 11.2	52.6 ± 12.2	59.4 ± 11.9	0.49
Ever-smoker	89 (30.5%)	2 (16.7%)	3 (37.5%)	0.88
Current drinker	70 (24.0%)	4 (33.3%)	2 (25.0%)	0.67
Excessive alcohol intake	2	0	0	1.00
BMI, kg/m^2^	26.8 ± 2.82	29.6 ± 5.17	29.7 ± 4.21	**<0.001**
BMI ≥27.5 kg/m^2^	99 (33.9%)	6 (50.0%)	6 (75.0%)	**0.009**
HOMA-IR	2.15 (1.63-2.93)	3.20 (2.38-4.63)	4.40 (2.65-7.15)	**<0.001**
Metabolic risk factors				
Central obesity	203 (69.5%)	12 (100.0%)	6 (75.0%)	0.16
Hypertension	181 (62.0%)	9 (75.0%)	7 (87.5%)	0.09
Prediabetes based on OGTT and/or HbA1c	187 (64.0%)	10 (83.3%)	8 (100%)	**0.01**
FG and/or HbA1c	165 (56.5%)	9 (75.0%)	5 (62.5%)	0.36
Low HDL-C or on lipid lowering medications	109 (37.3%)	5 (41.7%)	2 (25.0%)	0.65
High TG or on lipid lowering medications	145 (49.7%)	7 (58.3%)	5 (62.5%)	0.37
HOMA-IR ≥ 2.5	110 (37.7%)	9 (75.0%)	7 (87.5%)	**<0.001**
Number of metabolic risk factors	3.09 ± 1.45	4.25 ± 1.54	4.25 ± 1.16	**0.001**
Viral hepatitis B or C	16	0	1	1.00
ALT, U/L	25 (19-34)	38 (27-70)	43 (36-45)	**<0.001**
AST, U/L	24 (21-29)	28 (23-31)	44 (32-56)	**<0.001**
FIB4	1.03 (0.75-1.36)	1.28 (0.86-1.33)	1.68 (1.19-2.52)	**0.01**
NFS	-1.91 ± 1.24	-1.61 ± 1.17	-0.48 ± 0.88	**0.002**
CAP, dB/m	294.9 ± 33.7	303.8 ± 30.8	321.3 ± 37.6	**0.022**
LS, kPa	4.9 (4.2-5.6)	8.3 (8.1-8.6)	11.6 (10.4-20.4)	**<0.001**

Values expressed as mean ± standard deviation or median (25^th^ – 75^th^ percentile) or numbers (%). Cochran-Armitage test was applied for evaluating trend for binary response, whereas ANOVA linear test or Jonckheere-Terpstra test was applied for evaluating trend for continuous responses.

MAFLD, metabolic dysfunction-associated fatty liver disease; BMI, body mass index; OGTT, oral glucose tolerance test; HbA1c, glycated haemoglobin; FG, fasting glucose; HDL-C, high density lipoprotein-cholesterol; TG, triglyceride; HOMA-IR, homeostasis model assessment of insulin resistance; ALT, alanine aminotransferase; AST, aspartate aminotransferase; FIB-4, Fibrosis-4 index; NFS, non-alcoholic fatty liver disease fibrosis score; CAP, controlled attenuation parameter; LS, liver stiffness.

Bold values are those with statistical significance.

In multiple quantile regression analysis, in the first quantile, only BMI ≥27.5 kg/m^2^ (p=0.017) and central obesity (p=0.004) were significantly associated with LS, whereas in the second quantile, abnormal AST level became the only significant determinant (p <0.001). In the third quantile and the 90^th^ percentile, both abnormal AST level (p<0.001) and HOMA-IR ≥2.5 (p<0.001) were significant independent determinants of LS, in a model also consisting of hypertension, abnormal serum ALT level, prediabetes based on OGTT and/or HbA1c, as well as high TG or on lipid lowering medications. Moreover, the effects of abnormal AST and HOMA-IR ≥2.5 on LS increased with higher LS quantiles (**Table 3**).

**Table 3:** Multiple quantile regression analysis showing the independent determinants of higher liver stiffness in participants with overweight/obese MAFLD (N=312).

	25^th^ percentile		50^th^ percentile		75^th^ percentile		90^th^ percentile	
	Beta (95% CI)	p-value	Beta (95% CI)	p-value	Beta (95% CI)	p-value	Beta (95% CI)	p-value
BMI ≥ 27.5 kg/m^2^	0.40 (0.07 - 0.73)	**0.017**	0.30 (-0.04 - 0.64)	0.080	0.35 (-0.12 - 0.82)	0.146	0.38 (-0.56 - 1.31)	0.430
Central obesity	0.50 (0.16 - 0.84)	**0.004**	0.20 (-0.15 - 0.55)	0.263	0.45 (-0.04 - 0.94)	0.074	0.66 (-0.31- 1.64)	0.182
Abnormal AST level	0.40 (-0.26 - 1.06)	0.230	1.60 (0.93 - 2.27)	**<0.001**	1.53 (0.61 - 2.45)	**0.001**	3.92 (2.06 - 5.78)	**<0.001**
HOMA-IR ≥ 2.5	0.30 (-0.02 - 0.62)	0.066	0.30 (-0.03 - 0.63)	0.071	0.65 (0.20 - 1.10)	**0.005**	1.78 (0.87 - 2.69)	**<0.001**

The 25^th^, 50^th^, 75^th^ and 90^th^ quantiles corresponded to liver stiffness 4.3, 5.0, 5.8 and 7.0 kPa, respectively.

Model also included BMI, central obesity, hypertension, abnormal ALT level, prediabetes based on oral glucose tolerance test and/or HbA1c, high TG or on lipid-lowering medications.

MAFLD, metabolic dysfunction-associated fatty liver disease; 95%CI, 95% confidence interval; AST, aspartate aminotransferase; HOMA-IR, homeostasis model assessment of insulin resistance; BMI, body mass index; ALT, alanine aminotransferase; glycated haemoglobin; TG, triglyceride.

Bold values are those with statistical significance.

### Sequential screening algorithm for identifying individuals with non-diabetic overweight/obese MAFLD who had clinically significant liver fibrosis (LS ≥8.0 kPa)

Among these 312 participants with overweight/obese MAFLD and without diabetes, 20 (6.4%) of them had LS ≥8.0 kPa. Participants with LS ≥8.0 kPa had significantly higher BMI (p<0.001), abnormal ALT (p=0.038) and AST levels (p<0.001), NFS ≥-1.5 (p=0.003), prevalence of prediabetes based on OGTT and/or HbA1c levels (p=0.031) and HOMA-IR ≥2.5 (p=0.001) than those without (**Supplementary Table S1**).

To derive a screening algorithm for identifying individuals with overweight/obese MAFLD who had LS ≥8.0 kPa, multivariable stepwise logistic regression analysis was conducted in a stepwise fashion, based on the availability of parameters during the routine clinical care for patients with MAFLD. In the first step which consisted of BMI ≥27.5kg/m^2^, a cut-off used to define obesity among Asian individuals (19), as well as abnormal transaminase levels in the model, only abnormal AST level was independently associated with the presence of significant liver fibrosis (OR 8.96, 95%CI 3.3 – 24.3, p<0.001). In the next step when metabolic risk factors including prediabetes (based on OGTT and/or HbA1c) and HOMA-IR ≥2.5 were also included in the model, both abnormal AST level (OR 7.95, 95%CI 2.83 – 22.4, p<0.001) and HOMA-IR ≥2.5 (OR 5.01, 95%CI 1.54 – 16.3, p=0.008) remained independently associated with LS ≥8.0 kPa. (**Table 4**) The results were similar when abnormal serum transaminase levels were replaced by elevated NFS (**Supplementary Table S2**), or when BMI ≥27.5kg/m^2^ was replaced by a more generally used obesity cut-off of ≥30kg/m^2^. Notably, among the metabolic risk factors (**Supplementary Table S1**), HOMA-IR ≥2.5 was the only independent determinant of significant liver fibrosis (OR 4.08, 95%CI 1.54 – 16.0) in multivariable logistic regression analysis.

**Table 4:** Multivariable stepwise logistic regression showing the associations of clinical variables with LS ≥8.0 kPa in participants with overweight/obese MAFLD (N=312).

	OR (95% CI)	p-value
*Step 1: BMI*		
BMI ≥ 27.5 kg/m^2^	2.92 (1.16-7.39)	**0.023**
*Step 2: BMI, plus abnormal serum transaminase levels*		
BMI ≥ 27.5 kg/m^2^	2.43 (0.92-6.41)	0.07
Abnormal AST level	8.96 (3.30-24.3)	**<0.001**
*Step 3: BMI, abnormal serum transaminase levels, plus Metabolic risk factors*		
BMI ≥ 27.5 kg/m^2^	1.66 (0.60-4.60)	0.33
Abnormal AST level	7.95 (2.83-22.4)	**<0.001**
HOMA-IR ≥ 2.5	5.01 (1.54-16.3)	**0.008**

Abnormal serum transaminase levels included abnormal ALT and AST levels; Metabolic risk factors included presence of prediabetes based on oral glucose tolerance test and/or HbA1c, and HOMA-IR levels.

MAFLD, metabolic dysfunction-associated fatty liver disease; LS, liver stiffness; OR, odds ratio; 95%CI, 95% confidence interval; BMI, body mass index; AST, aspartate aminotransferase; HOMA-IR, homeostatic model assessment of insulin resistance; ALT, alanine aminotransferase; OGTT, oral glucose tolerance test; HbA1c, glycated haemoglobin.

Bold values are those with statistical significance.

### Performance of sequential screening algorithm in derivation and validation cohorts

**Figure 1:**
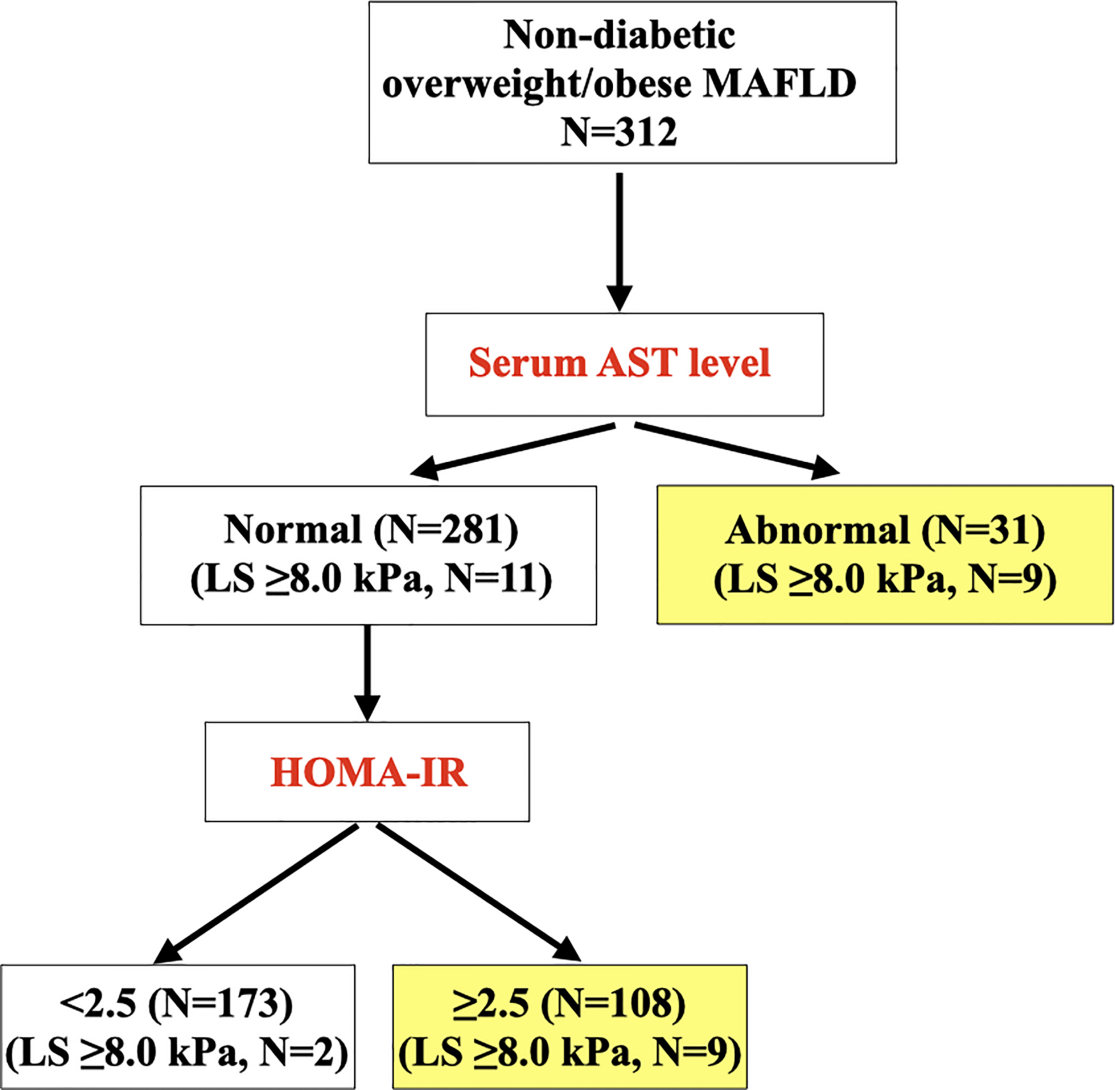
Sequential clinical algorithm for identifying overweight/obese participants with MAFLD at risk of clinically significant liver fibrosis (i.e. LS ≥8.0 kPa) (N=312). MAFLD, metabolic dysfunction-associated fatty liver disease; cACLD, compensated advanced chronic liver disease; LS, liver stiffness; AST, aspartate aminotransferase; HOMA-IR, homeostasis model assessment of insulin resistance.

In NCRISPS, the sensitivity and specificity of this sequential screening algorithm (**Figure 1**) to identify individuals with non-diabetic overweight/obese MAFLD who were at risk of clinically significant liver fibrosis was 90% and 58.6%, respectively. Importantly, the NPV was 98.8% with a PPV of 12.9%. (**Table 5**) The AUROC was 0.80 (95%CI 0.71 – 0.90). The performance was similar between men and women (**Supplementary Table S3**). In the pooled validation cohort, with baseline characteristics of the participants shown in **Supplementary Table S3**, the AUROC was 0.68 (95%CI 0.50 – 0.87). The sensitivity was 81.8% with an NPV of 91.3% (**Table 5**).

**Table 5:** Performance of the sequential screening algorithm in NCRISPS and the pooled validation cohort for identifying non-diabetic overweight/obese participants with MAFLD at risk of at risk of clinically significant liver fibrosis (i.e. LS ≥8.0 kPa).

	NCRISPS (Derivation cohort)	Pooled validation cohort
Sensitivity	18/20 (90.0%)	9/11 (81.8%)
Specificity	171/292 (58.6%)	20/60 (35.0%)
PPV	18/139 (12.9%)	9/48 (18.8%)
NPV	171/173 (98.8%)	21/23 (91.3%)

NCRISPS, New cardiovascular risk factor prevalence study; MAFLD, metabolic dysfunction-associated fatty liver disease; LS, liver stiffness; PPV, positive predictive value; NPV, negative predictive value.

## Discussion

In this contemporary population-based study of HK Chinese with comprehensive metabolic evaluation, we demonstrated that 1 in 3 of our local community-dwelling individuals had overweight/obese MAFLD. However, despite this high prevalence, only <3% and 6.4% of them had advanced and clinically significant liver fibrosis, respectively. Therefore, we have developed a simple, sequential screening algorithm based on abnormal AST, followed by elevated HOMA-IR levels. We demonstrated, with external validation, that the clinical performance of this algorithm was satisfactory with sensitivity over 80% and NPV over 90%. Although the specificity and PPV were relatively low, the high sensitivity and NPV were particularly important for a screening algorithm, which allowed us to optimally identify, among this large group of individuals with non-diabetic overweight/obese MAFLD, those who were at risk of cACLD and would require referral to hepatologists for VCTE and/or further hepatic evaluation.

Since the proposal of the new diagnostic entity of MAFLD, only a few studies have directly compared the three different MAFLD subgroups (9–11). The Rotterdam Study showed that the prevalence of hepatic fibrosis, defined as LS ≥8.0 kPa, increased significantly when individuals fulfilled all three diagnostic criteria of MAFLD which included type 2 diabetes, overweight/obesity or having two or more metabolic abnormalities, as compared to those satisfying only one or two inclusion criteria (23). It is also well established that type 2 diabetes is an important risk factor of fibrosis progression in fatty liver disease (24). A recent meta-analysis reported that 1 in 5 patients with type 2 diabetes had elevated LS (25). Consistently, in our study, individuals with T2D-MAFLD had significantly higher LS and prevalence of clinically significant liver fibrosis than the other two MAFLD subgroups. Indeed, several non-invasive fibrosis scores and novel biomarkers have been investigated over the years for their performance to stratify liver fibrosis risk specifically among individuals with T2D-MAFLD (26–28). (29) On the other hand, as shown by us and others, non-diabetic overweight/obese MAFLD constitutes the largest MAFLD population within the community (9–11). Although the studies were not directly comparable, our 73% prevalence of non-diabetic overweight/obese MAFLD was overall similar to the 77.5% reported in a community-based survey in Beijing, and lower than the 95.2% in a Korean study (9, 10). However, we found that their overall risk of significant liver fibrosis was much lower than that of T2D-MAFLD and correlated significantly with the presence of additional metabolic comorbidities.

Hence, in this study, we evaluated whether taking into consideration the presence of additional metabolic risk factors would improve the identification of clinically significant liver fibrosis specifically among individuals with overweight/obese MAFLD. The contemporary study population, together with the comprehensive metabolic assessments, which included OGTT in all our participants (except for those taking anti-diabetic medications), are two major strengths of our study. Indeed, we found that 41.7% of our study participants had MAFLD, a prevalence rate that was considerably higher than the 25.9% reported in a local population study using the HK census database performed over a decade ago (30). Although the two studies differed in the imaging modality employed for the detection of hepatic steatosis, our updated local MAFLD prevalence was overall in keeping with that reported globally in a recent meta-analysis (2). This probably reflected the soaring prevalence of obesity and related metabolic diseases such as prediabetes both locally and globally (31, 32), and the additional use of OGTT for evaluating glycaemic status in our study.

We found that, of all the metabolic risk factors including obesity, central adiposity, hypertension, dyslipidaemia and prediabetes diagnosed based on OGTT and/or HbA1c, elevated HOMA-IR was the only independent determinant of LS >8.0 kPa. These findings, which were derived from non-diabetic overweight/obese MAFLD participants, concurred with those reported in a previous study of obese individuals with NAFLD that HOMA-IR was an independent predictor of worsening histological fibrosis (33). Indeed, amongst the multiple hits in the pathogenesis of fatty liver disease, insulin resistance is a key driver of its progression (34). With increased lipolysis in the adipocytes and *de novo* lipogenesis in the liver, free fatty acid accumulates and lipo-toxicity ensues. Hepatocyte injury causes inflammation with increased cytokines production by the Kupffer cells, vascular remodeling, and activation of regenerative processes. Repetitive unsuccessful regenerative responses lead to progressive scarring, advanced fibrosis and cirrhosis (35). Furthermore, hyperinsulinaemia, which occurs secondary to insulin resistance, also promotes hepatic fibrosis through stimulating the proliferation of hepatic stellate cells, collagen synthesis, and up-regulation of the hepatic expression of connective tissue growth factors (36, 37).

In this study, with the inclusion of OGTT for metabolic evaluation in our study, we found that both HOMA-IR≥2.5 and prediabetes based on either OGTT or HbA1c were important metabolic risk factors of liver fibrosis among individuals with non-diabetic overweight/obese MAFLD. However, our findings in multivariable analyses showed that elevated HOMR-IR outperformed prediabetes, which included also individuals with normal HbA1c but abnormal OGTT, a cumbersome test to perform. This led to the development of the current screening algorithm as a simpler strategy to use clinically, based on parameters that can be conveniently measured during the routine care for patients with MAFLD. On the other hand, we found that FIB-4, a commonly used non-invasive fibrosis score, was not significantly associated with LS ≥8.0 kPa, which was likely due to the low prevalence of liver fibrosis in this community-based cohort. Moreover, although we demonstrated that replacing abnormal AST level with NFS resulted in similar conclusions, it is noteworthy that NFS is a composite score that requires several other parameters including platelet count, serum albumin and ALT levels. Certainly, the relatively small sample size of the two external cohorts to validate our findings was a major limitation of the study. Nonetheless, we found that the clinical performance of this screening algorithm remained satisfactory in the pooled validation cohort with reasonable sensitivity and high NPV of over 90%, indicating that this algorithm should be also applicable to individuals who are relatively insulin resistant either due to PCOS or more severe obesity.

Our study had several other limitations. First, the cross-sectional study design precluded the evaluation of a causal relationship between metabolic dysfunction and the development of liver fibrosis, or cACLD, in patients with overweight/obese MAFLD. Secondly, the sample size of both derivation and validation cohorts were relatively small, and all our participants were HK Chinese. Further studies in other populations with a larger sample size are required to confirm these findings and validate our proposed clinical algorithm. Moreover, serum hsCRP level was not measured and liver biopsy was not performed in our study participants. However, it was not feasible and ethically not justified to perform liver biopsy in these asymptomatic community-dwelling individuals. Nonetheless, the prevalence of 6.4% with LS ≥8.0 kPa on VCTE in our study, which involved community-dwelling individuals aged ≥25 years, was overall in line with those reported in two recent Korean studies based on magnetic resonance elastography (9, 10). In these two studies conducted among individuals attending health check-up aged ≥18 years and ≥40 years, the prevalence of significant liver fibrosis was found to be 4.2% and 9.9%, respectively (9, 10). Lastly, although the recruitment process from the community was through random sampling, participation in this population-based study was entirely voluntary. Therefore, it is possible that the participants were overall relatively more health conscious, which could have also explained the small number of individuals with excessive alcohol intake that is associated with increased liver fibrosis development in MAFLD (38). Moreover, the relatively small sample size of individuals with viral hepatitis or the significant alcohol intake also rendered it difficult for further subgroup analysis based on MAFLD with single or dual etiologies.

## Conclusion

It is conceivable that a MAFLD pandemic, in particular overweight/obese MAFLD, will soon follow alongside the rising global prevalence of obesity (2, 39). From a clinical perspective, our findings suggest the recommendations of measuring serum AST level in all individuals with non-diabetic overweight/obese MAFLD detected on imaging techniques such as ultrasound or blood biomarkers, and have serum fasting insulin level measured to determine the HOMA-IR if their serum AST levels are normal. Individuals who have elevated serum AST level and/or HOMR-IR ≥2.5 should be referred for VCTE and/or hepatologist assessment for the presence of clinically significant liver fibrosis. Since MAFLD research has only started since 2020, future prospective studies should focus on the role of metabolic dysfunction in stratifying the long-term risks of incident adverse hepatic outcomes including liver-related mortality in this largest subgroup within the MAFLD population.

## Data availability statement

The datasets generated during and/or analysed during the current study are available from the corresponding author on reasonable request.

## Ethics statement

The studies involving human participants were reviewed and approved by Ethics Committee of the University of Hong Kong/Hospital Authority Hong Kong West Cluster (IRB Ref: UW-18-610). The patients/participants provided their written informed consent to participate in this study.

## Author contributions

C-HL researched the data and wrote the manuscript. DL, MY, RL, LM and Y-CW researched the data. CF, AL and JC performed statistical analyses. T-HL, JW, AX, H-FT, KT, BC, M-FY and KL critically reviewed and edited the manuscript. KL initiated and supervised the study, had full access to all the data and took responsibility for the integrity of the data and the accuracy of the data analysis. All authors contributed to the article and approved the submitted version.
