# Supplementary material for: Sequential algorithm to stratify liver fibrosis risk in overweight/obese metabolic dysfunction-associated fatty liver disease
Source: Front Endocrinol (Lausanne). 2023 Jan 6;13:1056562. doi: 10.3389/fendo.2022.1056562 (PMC9853017; doi:10.3389/fendo.2022.1056562)
Supplement: Supplementary file 1 [file Table_1.docx]

Supplementary Table S1 Clinical characteristics of participants with overweight/obese MAFLD by the presence of clinically significant liver fibrosis (i.e. LS ≥8.0 kPa) (N=312)

|  | LS <8.0 kPa | LS ≥8.0 kPa | Unadjusted OR (95% CI) | p-value |
| --- | --- | --- | --- | --- |
| N | 292 | 20 | -- | -- |
|  |  |  |  |  |
| *Demographic and*  *anthropometric parameters* |  |  |  |  |
| Men | 161 (55.5%) | 13 (65%) | 1.49 (0.58-3.84) | 0.41 |
| Age, years | 54.7±11.2 | 55.3±12.3 | 1.01 (0.97-1.05) | 0.79 |
| Ever-smoker | 88 (30.3%) | 5 (25%) | 0.76 (0.37-2.16) | 0.76 |
| Current drinker | 70 (24.1%) | 6 (30%) | 1.36 (0.50-3.67) | 0.55 |
| Excessive alcohol intake | 2 (0.68%) | 0 (0%) | - | - |
| BMI, kg/m^2^ | 26.8±2.81 | 29.6±4.69 | 1.25 (1.11-1.41) | **<0.001** |
| BMI ≥ 27.5 kg/m^2^ | 99 (34.1%) | 12 (60%) | 2.92 (1.16-7.39) | **0.02** |
|  |  |  |  |  |
| *Liver parameters* |  |  |  |  |
| Viral hepatitis B or C | 16 | 1 | 0.91 (0.11-7.22) | 0.927 |
| ALT, U/L | 26 (19-36) | 42 (31-54) | 1.03 (1.01-1.05) | **0.002** |
| Abnormal ALT | 28 (9.7%) | 5 (25%) | 3.14 (1.06-9.30) | **0.04** |
| AST, U/L | 24 (21-29) | 32 (28-50) | 1.09 (1.05-1.13) | **<0.001** |
| Abnormal AST | 22 (7.6%) | 9 (45%) | 10.0 (3.76-26.8) | **<0.001** |
| FIB-4 ≥1.45 | 63 (21.7%) | 7 (35%) |  |  |
| FIB-4 |  |  |  |  |
| <1.45 | 227 (78.3%) | 13 (65%) |  |  |
| 1.45 – 3.25 | 63 (21.7%) | 6 (30%) |  |  |
| >3.25 | 0 | 1 (5%) | 1.96 (0.75-5.11) | 0.17 |
| NFS |  |  |  |  |
| < -1.5 | 179 (61.7%) | 5 (25%) |  |  |
| -1.5 to <0.67 | 110 (37.9%) | 14 (70%) |  |  |
| ≥0.67 | 1 (0.3%) | 1 (5%) |  |  |
| NFS ≥-1.5 | 111 (38.3%) | 15 (75%) | 4.89 (1.73-13.8) | **0.003** |
|  |  |  |  |  |
| *Metabolic risk factors* |  |  |  |  |
| Central obesity | 203 (70%) | 18 (90%) | 3.88 (0.88-17.0) | 0.07 |
| Hypertension | 181 (62.4%) | 16 (80%) | 2.41 (0.79-7.39) | 0.12 |
| Prediabetes based on  OGTT and/or HbA1c | 185 (63.8%) | 18 (90%) | 5.11 (1.16-22.4) | **0.03** |
| FG and/or HbA1c | 163 (56.2%) | 14 (70%) | 1.82 (0.68-4.86) | 0.23 |
| Low HDL-C or on lipid-lowering medications | 109 (37.6%) | 7 (35%) | 0.89 (0.35-2.31) | 0.82 |
| High TG or on lipid-lowering medications | 145 (50%) | 12 (60%) | 1.50 (0.60-3.78) | 0.39 |
| HOMAIR ≥2.5 | 110 (37.9%) | 16 (80%) | 6.55 (2.13-20.1) | **0.001** |

Values expressed as mean±standard deviation or median (25^th^ – 75^th^ percentile) or numbers (%).

MAFLD, metabolic dysfunction-associated fatty liver disease; LS, liver stiffness; OR, odds ratio; 95%CI, 95% confidence interval; BMI, body mass index; ALT, alanine transaminase; AST, aspartate aminotransferase; FIB4, fibrosis-4 index; NFS, NAFLD fibrosis score; OGTT, oral glucose tolerance test; HbA1c, glycated haemoglobin; FG, fasting glucose; HDL-C, high density lipoprotein-cholesterol; TG, triglyceride; HOMA-IR, homeostasis model assessment of insulin resistance.

Supplementary Table S2 Multivariable stepwise logistic regression showing the associations of clinical variables, including NFS instead of transaminase levels, with the presence of clinically significant liver fibrosis (i.e. LS ≥8.0 kPa) in participants with overweight/obese MAFLD (N=312)

|  | OR (95% CI) | p-value |
| --- | --- | --- |
| *Step 1:*  *Demographic and anthropometric parameters* |  |  |
| BMI ≥ 27.5 kg/m^2^ | 2.92 (1.16-7.39) | **0.02** |
|  |  |  |
| *Step 2:*  *Demographic and anthropometric parameters, plus NFS* |  |  |
| BMI ≥ 27.5 kg/m^2^ | 2.84 (1.10-7.29) | **0.03** |
| NFS ≥ -1.5 | 4.79 (1.68 – 13.6) | **0.003** |
|  |  |  |
| *Step 3:*  *Demographic and anthropometric parameters, plus*  *NFS, plus*  *Metabolic risk factors* |  |  |
| BMI ≥ 27.5 kg/m^2^ | 1.95 (0.73 – 5.22) | 0.18 |
| NFS ≥ -1.5 | 4.69 (1.62 – 13.6) | **0.004** |
| HOMA-IR ≥ 2.5 | 5.50 (1.73 – 17.5) | **0.004** |

Demographic and anthropometric parameters included BMI; Metabolic risk factors included the presence of prediabetes based on oral glucose tolerance test and/or HbA1c, and HOMA-IR levels.

MAFLD, metabolic dysfunction-associated fatty liver disease; LS liver stiffness; OR, odds ratio; 95%CI, 95% confidence interval; BMI, body mass index; HOMA-IR, homeostatic model assessment of insulin resistance; NFS, non-alcoholic fatty liver disease fibrosis score; HbA1c, glycated haemoglobin.

Supplementary Table S3 Performance of the sequential screening algorithm in NCRISPS for identifying non-diabetic overweight/obese participants with MAFLD at risk of at risk of clinically significant liver fibrosis (i.e. LS ≥8.0 kPa) with participants stratified by sex

|  | NCRISPS (Derivation cohort) | | |
| --- | --- | --- | --- |
|  | All | Men | Women |
| N | 312 | 175 | 137 |
| Sensitivity | 18/20 (90.0%) | 11/13 (84.6%) | 7/7 (100%) |
| Specificity | 171/292 (58.6%) | 97/162 (59.9%) | 74/130 (56.9%) |
| PPV | 18/139 (12.9%) | 11/76 (14.5%) | 7/63 (11.1%) |
| NPV | 171/173 (98.8%) | 97/99 (98.0%) | 74/74 (100%) |

NCRISPS, New cardiovascular risk factor prevalence study; MAFLD, metabolic dysfunction-associated fatty liver disease; LS, liver stiffness; PPV, positive predictive value; NPV, negative predictive value.

Supplementary Table S4 Clinical characteristics of the participants with non-diabetic overweight/obese MAFLD in NCRISPS and the pooled validation cohort

|  | Pooled validation cohort | NCRISPS |
| --- | --- | --- |
| N | 71 | 312 |
| Women | 57 (80.3%) | 137 (43.9%) |
| Age, years | 38.5±9.55 | 54.5±11.2 |
| BMI, kg/m^2^ | 31.8±5.13 | 26.9±3.04 |
| BMI ≥27.5kg/m^2^ | 58 (81.7%) | 111 (35.6%) |
| ALT, U/L | 25 (18-43) | 26 (19-37) |
| Abnormal ALT | 19 (26.8%) | 33 (10.6%) |
| AST, U/L | 25 (19-29) | 25 (21-29) |
| Abnormal AST | 12 (16.9%) | 31 (9.9%) |
| HOMA-IR ≥2.5 | 46 (64.8%) | 126 (40.4%) |
| CAP, dB/m | 311±42.7 | 296±34 |
| LS, kPa | 5.4 (4.4-6.7) | 5.0 (4.3-5.8) |
| LS ≥ 8.0 kPa | 11 (15.5%) | 20 (6.4%) |

PCOS, polycystic ovarian syndrome; BMI, body mass index; ALT, alanine aminotransferase; AST, aspartate aminotransferase; HOMA-IR, homeostasis model assessment of insulin resistance; CAP, controlled attenuation parameter; LS, liver stiffness.
